# Powdered Cellulose Microblasting for Dry Cleaning Printed Works on Paper

**DOI:** 10.3390/polym16020176

**Published:** 2024-01-07

**Authors:** Iris Bautista-Morenilla, Cristina Ruiz-Recasens, Gema Campo-Francés

**Affiliations:** Research Group of Conservation of Cultural Heritage, Art and Conservation Department, Fine Arts Faculty, University of Barcelona, Pau Gargallo, 4, 08028 Barcelona, Spain; cruiz@ub.edu (C.R.-R.); gcampo@ub.edu (G.C.-F.)

**Keywords:** cellulose powder, cultural heritage, cleaning methods, microblasting, paper artwork

## Abstract

This study evaluates the practical feasibility of using powdered cellulose microblasting for dry cleaning paper-based printed artworks in a real setting of conservation treatment. The control parameters used for this purpose are the potential morphological changes in the surface, the level of cleanliness achieved, and the amount of residue remaining in the artwork after the treatment. In this study, cleaning of a lithography was conducted entirely with powdered cellulose microblasting. The outcomes were evaluated before and after treatment using optical microscopy, scanning electron microscopy, energy-dispersive X-ray spectroscopy, and spectrophotometry. The results indicate that powdered cellulose microblasting is a feasible and efficient technique for conducting the dry cleaning of printed works on paper without causing morphological changes to their surface. Additionally, it offers significant benefits by enabling precise treatment control, reducing cleaning time, and using materials stable in the long term and compatible with the substrate. Moreover, it mitigates the long-term negative effects caused by synthetic polymer residues from the cleaning materials commonly used in the dry cleaning of paper.

## 1. Introduction

Dry cleaning is a conservation treatment often conducted on cultural assets for the removal of unwanted materials such as surface soil, dust, dirt, insect droppings, build-up, or other surface deposits [1]. Depending on the characteristics of the work and the specific circumstances of each case, this treatment may be considered necessary for preventive reasons, due to the conservation risks associated with certain deposits on the surface of the object, or for aesthetic purposes, in order to improve the legibility of the artefact. However, dry cleaning of paper-based works can be a disruptive process that may cause abrasion of the paper surface, forcing foreign materials such as dust or eraser powder into the paper matrix and damaging the media [2]. Indeed, cleaning remains a complex conservation challenge that requires enhanced research and practical efforts to develop and evaluate a range of new or modified options [3,4]. Methods typically used to conduct this treatment on paper-based artworks and documents have been shown to have significant disadvantages for the artefact’s characteristics and its long-term stability [5,6].

Vacuum cleaning is commonly used as the initial phase of a dry cleaning procedure and is considered one of the least intrusive methods. Often, it is complemented by the use of a soft brush to optimize results. However, its effectiveness is limited or insignificant when dirt has solidified due to biological, physical, and chemical processes [7,8,9].

Erasers and sponges are widely used for dry cleaning documents, offering a significant benefit in terms of ease of control. The cleaning process can be halted as needed, enabling the exclusion of fragile areas or regions that do not require cleaning. Both erasers and sponges demonstrate effective removal of visible dirt between fibers. Additionally, these techniques are cost-effective and do not necessitate the use of specialized equipment [10]. However, the literature highlights their main drawbacks [11,12,13,14,15] related to the stability of the media, changes in brightness and/or surface texture [16,17], as well as residues that, to a greater or lesser extent depending on the type of the treated paper, infiltrate the interstices of the paper fibers [18], affecting the absorption properties of the artwork [19,20] and posing a risk to the future conservation of the work due to containing abrasive materials, sulfur, hydrochloric acid, plasticizers, drying oils, etc. [21,22,23,24].

Microblasting is another alternative to dry cleaning. Its efficiency, effectiveness, and fundamental processes in the cleaning of historic surfaces have been reported in the literature [6,25]. Microblasting involves projecting abrasive particles propelled by pressurized air to disrupt the adhesion between surface deposits and the substrate, a phenomenon based on the kinetic energy formula (E_k_ = ½m × v^2^), where m is mass or abrasive, and v is velocity [26]. The scientific literature details various application parameters, with the most influential factors including pressure, distance, angle, time, nozzle, particle flow, and specific abrasive properties. Properly selecting the abrasive and adjusting these key parameters can thereby alter the effects on the surface, where the least aggressive angle for different substrates is close to 75° [25,26,27,28].

Although microblasting has been mainly used in architectural preservation [29], previous scientific studies have obtained positive experimental results in paper works [30,31,32] and canvas paintings [10,33] by replacing hard abrasives traditionally used in inorganic supports with high-purity powdered cellulose microfibers, a VOC/SVOC-free sulfite bleached pulp from nonconiferous wood [33]. A fiber length below 40 µm has been found to produce successful results in previous experimental dry cleaning studies [10,30,31,32]. Indeed, microblasting of powdered cellulose emerged as an advantageous system due to its higher cleaning efficiency without affecting the morphology of the surfaces, and the low amount of cleaning material residue remaining in the treated samples. These included porous papers, even those in poor condition with low mechanical properties, open fiber structure, and rough surface texture.

However, so far, microblasting with powdered cellulose has been only conducted in selected spots in limited extent. Its applicability to the conservation treatment of an artefact remains uncertain and may present unforeseen challenges inherent to the transfer of an experimental method to the real-world context.

On this basis, the authors hypothesized that microblasting of powdered cellulose could be an alternative to conventional dry cleaning methods, avoiding their proven negative effects and enhancing treatment development. The authors of this study and other authors in the literature [6] consider powdered cellulose to be suitable for cleaning paper-based works due to its compatibility with the support and long-term stability. Compatibility ensures that the cleaning material does not cause any adverse reactions with the original work, including the paper, inks, or other constituent elements. Long-term stability makes it appropriate for use in conservation projects, complying with one of the mandatory principles in the field.

In the view of the above, this research aimed at evaluating the practical feasibility and the effectiveness of powdered cellulose microblasting for dry cleaning paper within the real context of a conservation treatment. The control parameters used for this purpose were the potential morphological changes in the surface, the level of cleanliness achieved, and the amount of residue remaining in the artwork after the treatment. The treatment’s performance was evaluated based on factors commonly considered in conservation projects. These include the device’s ease of use in cleaning large surfaces, the time required to complete the treatment, the ability to adjust the cleaning intensity in real time based on the treatment needs and specificities, the capacity to stop the cleaning promptly in unexpected circumstances, and the technique’s spatial resolution, which enables the treatment of specific areas without affecting their surroundings.

## 2. Materials and Methods

The methodology involved applying the cleaning system to a paper artwork with surface dirt. Various parameters were measured at different locations before and after treatment using optical microscopy, electron microscopy, and spectrophotometry.

**Figure 1:**
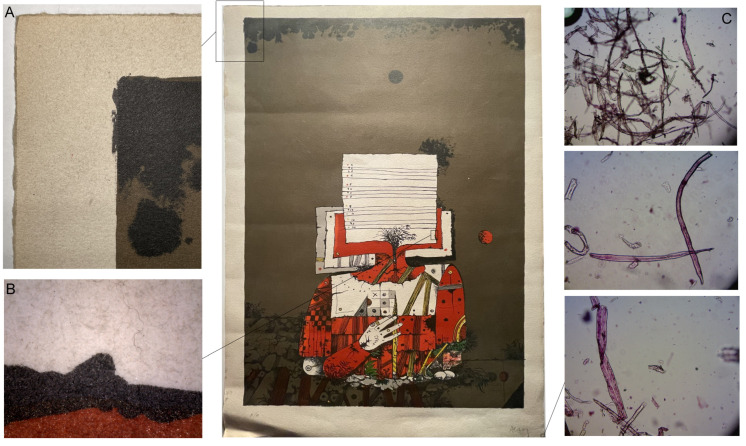
Selected lithograph for the study. (**A**) Margin detail: The paper appears to have a porous and loosely packed fiber structure. The large amount of surface dirt is visible to the naked eye. (**B**) Central area detail: The contrast between the surface of inked areas and the support is visible. In the red ink, less penetration of dirt is especially clear due to the smoother texture of the inked areas. The gloss effect created by overlapping of the inks, red and black in this case, is also apparent. (**C**) Optical microscopy fiber analysis with Lofton–Merrit staining test: results indicate that paper is made of sulfite-based bleached chemical pulp with some unbleached fibers. The fibers are in an acceptable condition. The small amount of sizing is apparent from the minimal visible residue between fibers and the ease of separation without prewashing.

The selected artwork was a sacrificial work, an artist’s proof lithograph created approximately 20 years ago on a 220 g/m^2^; intaglio paper with a relatively smooth texture, although the surface was quite porous due to low sizing (Figure 1).

The support was a bleached sulfite-based chemical pulp paper with some unbleached fibers. The mechanical strength of the artwork was good, and its fibers were in good condition. However, due to the low degree of sizing, it was mechanically sensitive to abrasion or friction and was rather porous.

Prior to treatment, the primary concern with the paper was the substantial accumulation of dust and surface dirt that had infiltrated the interstices of the surface fibers. The ink exhibited a comparable level of dust, but due to the smoother and more continuous surface of lithographic ink, particles remained more superficial.

**Figure 2:**
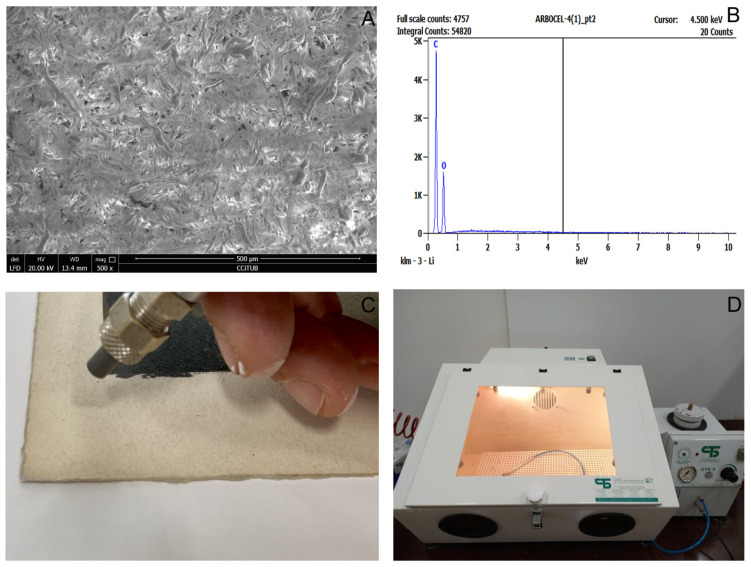
At the top, SEM image of powdered cellulose Arbocel^®^ at 500× magnification (**A**) and the EDX related spectra (**B**). At the bottom, the equipment used for cleaning is observed: picture C shows a straight tungsten carbide nozzle with a diameter of 0.7 mm, and picture (**D**) shows a footswitch-operated microblaster, a CTS5/B, and a Box CTS4 cabinet with an environmental dust collector.

The cleaning was carried out using Arbocel^®^ BE600-30PU (Barcelona, Spain) cellulose with a particle size of 30 µm from J. Rettenmaier and Söhne (Figure 2). According to the literature, this fiber size is the least damaging to paper surfaces [6] and acrylic inks [32]. Cellulose fibers were projected using a CTS5/B (CTS Europe, Barcelona, Spain) footswitch-operated microblaster through a straight tungsten carbide nozzle with a diameter of 0.7 mm. A silenced 1.5 HP compressor and a dehumidifier filter (CTS Europe, Barcelona, Spain) were also employed to reduce the compressed air’s humidity, preventing cellulose clumping.

The treatment was developed in a Box CTS4 cabinet (CTS Europe, Barcelona, Spain) with an environmental dust collector. The cleaning process was carried out by drawing circles across the entire surface of the work to ensure homogeneity and prevent unwanted marks, as recommended in the literature [6]. Cleaning parameters were selected based on previous studies and were tested before conducting the cleaning trials to identify any necessary adjustments. The pressure (20 kPa) and angle (70°) were not modified. Distance and time were adjusted and reduced when it was determined that cleaning was complete for each area of the artwork.

In previous research [26,30], 20 kPa has been demonstrated to produced the highest cleaning efficiency with paper substrates without causing mechanical damage to the treated surfaces. Additionally, a working angle of 70° has been shown to minimize the cleaning mechanism via impact, which is inherent to right angles, while enhancing friction or cutting mechanisms. This typically results in milder effects on the surface [26].

Optical microscopy, scanning electron microscopy, and spectrophotometry analyses were conducted before and after the cleaning treatment to verify its feasibility without causing morphological surface changes and to detect any residues of cleaning material on the artefact.

**Figure 3:**
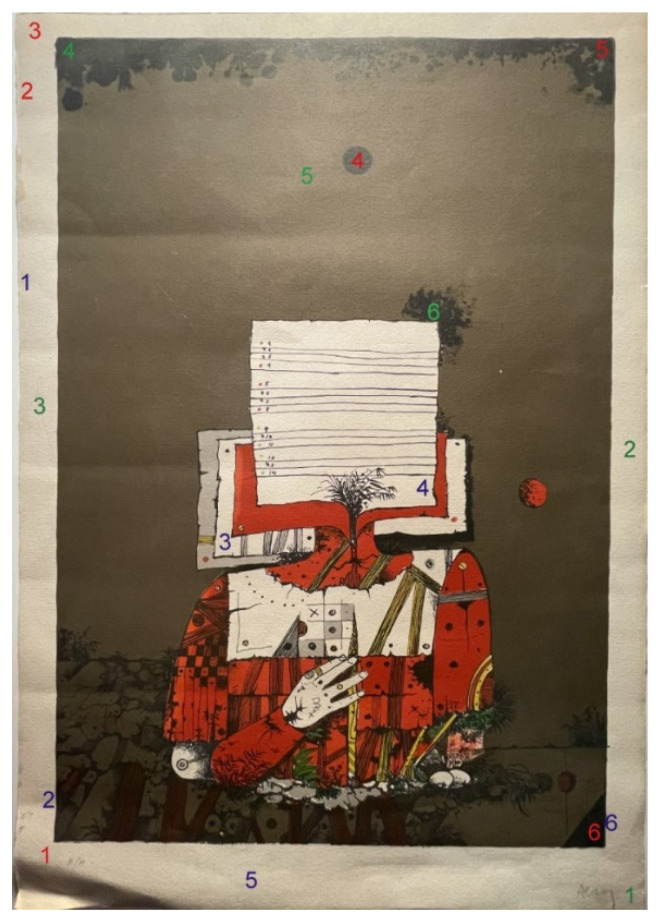
Location of the spots examined with each analytical technique: optical microscopy (marked in blue), scanning electron microscopy (market in red), and spectrophotometry (marked in green).

The artwork underwent examination in six representative areas using optical microscopy. Six samples were extracted for analysis through scanning electron microscopy, and six spectrophotometry readings were taken—three in the paper area to assess the level of cleanliness and three in the black ink area to evaluate residues (Figure 3).

Optical microscopy was performed using a AM4113-FVW Dino-Lite^®^ (AnMo Electronics Corporation, Getafe, Spain) surface microscope at magnifications ranging from 60× to 200× with direct LED. All the images were processed with 2.0 Dino-Capture^®^ software. Analysis of surface morphology allowed the detection of possible texture changes or fiber alterations, accurately determining the effects and effectiveness of the cleaning tests.

Scanning electron microscopy was carried out using an FEI ESEM QUANTA 200 (FEI Company, Barcelona, Spain) instrument in low-vacuum mode. The analyses were conducted in a vacuum chamber at 130 Pa with an acceleration voltage of 20 kV and a working distance between 9.4 and 10.4 mm. Images were captured in backscattered electron mode, and the morphology of the samples was studied at magnifications between 100× and 2000×. Determining the surface morphology before and after cleaning enabled the identification of possible texture changes, any residues remaining on the surface, and a more precise assessment of the effectiveness of the tested cleaning system.

A Konica Minolta CM 2600d spectrophotometer (Konica Minolta, Valencia, Spain) with a range of 400 to 700 nm and a measurement interval of 10 nm was used to assess potential color changes resulting from the cleaning process. Analyses were conducted with a 10° reflection optical geometry and a measurement area of Ø 5 mm. The results are expressed in the CIELAB 1976 system (Konica Minolta, Valencia, Spain) with reference to illuminant D65.

The chromatic values were obtained using CM-S100w 3.20.0002 Spectra Magic software (Konica Minolta, Valencia, Spain) and processed in a spreadsheet to obtain the differences in each of the three coordinates (ΔL*, Δa*, and Δb*) before and after cleaning.

## 3. Results and Discussion

Surface optical microscopy at 120× magnification indicates that the results meet the feasibility control parameters established at the beginning of the study. The evidence shows that the level of cleanliness achieved was remarkable, even in the spaces between fibers. Additionally, no morphological changes or residues were detected in the artwork after the treatment.

**Figure 4:**
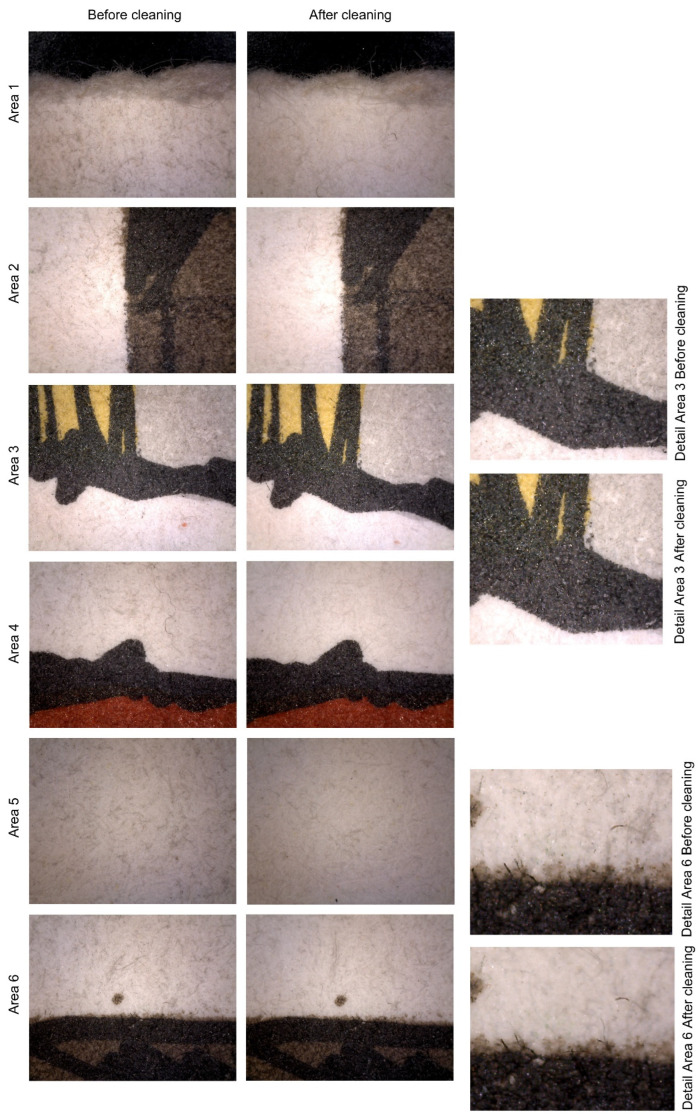
Photographs from optical microscopy taken at 120× magnification before treatment (**left column**) and after cleaning (**right column**). The numbers in each photograph correspond to the area that was examined, as located in Figure 3. Also, areas 3 and 6 are shown in detail on the right.

Figure 4 displays six areas that were studied before (left) and after (right) cleaning. The selected spots included both media and noninked paper to test the cleaning results on the support and ink. The results show that the paper became whiter after cleaning, while the intensity of the inks remained unchanged. Additionally, no surface changes were detected in any of the treated areas, and no residues were observed at 120× magnification.

Prior to treatment, the surfaces showed dirt particles of two different sizes, circular in shape and brown to black in color, especially visible in noninked areas (refer specially to images of areas 1 and 5 in Figure 4). There was also dirt of smaller granulometry that had penetrated the interfiber spaces, visually resulting in a continuous medium grey tone in some areas. After cleaning, nearly all circular particles was removed, and interfiber dirt was reduced. As a result, the paper had a much lighter appearance. In the detailed view of area 6 (Figure 4), it is clear that, after cleaning, the circular particles remaining on the surface were negligible. The grey tone of the interfiber soiling was reduced, and no residues of the powdered cellulose used in the treatment were visible. Additionally, it was evident that the fibers of the paper maintained their position, even those that were free, without ink to consolidate them to the more compacted base of the support. This is consistent with the results previously reported in the literature [6,30], where this technique was found to be effective in removing dirt from paper without damaging the surface.

As previously mentioned, the visual intensity of the inked areas remained the same, indicating that no visual loss of color materials was detected. This is in accordance with what has been reported in the literature, both for etching inks [31] and acrylics [32]. Yellow- and red-colored areas became more vivid after cleaning (refer to areas 3 and 4, Figure 4), and grey ink was perceived lighter (see areas 2 and 3 in Figure 4). Black ink appeared deeper and stronger, creating a greater contrast with the colors and the background. The inks also exhibited the same level of brightness, maintaining the nuances of transparency and changes in reflectance when overlapping (refer to the detail of area 3 in Figure 4).

As for residues, the black ink areas also show no traces of cellulose powder residue, which would appear as visible lumps due to their white color, as reported in previous studies [31].

The electron microscopy confirmed the same observations regarding morphology, cleaning efficiency, and absence of residues. Similar results were obtained, with unnoticeable changes in surface morphology, remarkable removal of soiling, and no residues of the cleaning material detected at 2000× magnification.

**Figure 5:**
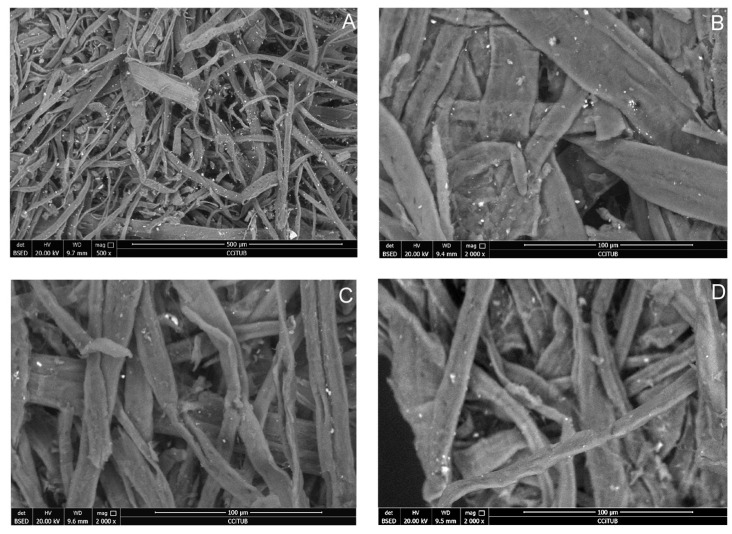
Photographs from electron microscopy taken at 500× (**A**) and at 2000× magnification (**B**–**D**), before treatment (upper image (**A**,**B**)) and after cleaning (lower image (**C**,**D**)). These images show samples taken from the upper left corner of the stamp (spot (**C**), marked in red in Figure 3).

Figure 5 shows the studied area before and after cleaning. The upper images display twisted and contoured document fibers, along with numerous white or light grey dirt particles. In contrast, the lower images show the paper after cleaning, with fewer dirt particles and the same fiber morphology as before cleaning. No residues of powdered cellulose were observed, which would be clearly visible at this magnification, as previously reported [10].

**Figure 6:**
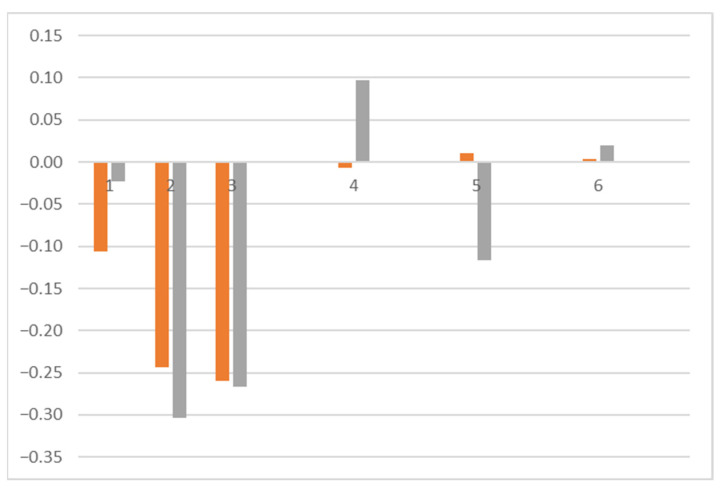
Difference in a* (orange) and b* (grey) at each measured point, before and after cleaning.

**Figure 7:**
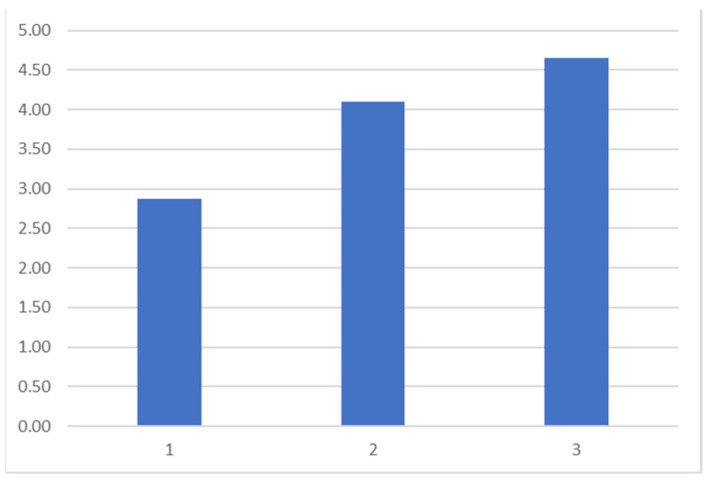
Spots 1, 2, and 3: difference in L* in measurements on the support of the artwork without the presence of supporting elements, before and after cleaning.

The cleaning efficacy previously detected using optical microscopy was confirmed via spectrophotometry, which revealed the effects of cleaning on the color of the artwork. In Figure 6 and Figure 7, we can observe the difference in L* (blue), a* (orange), and b* (grey) at different measured spots before and after cleaning.

Spots 1, 2, and 3 were readings performed on the paper without ink on it (refer to Figure 3 to locate the measured areas). The difference in a* and b* (Figure 6) and the difference in L* (Figure 7) indicate that after cleaning, the paper became less reddish-yellow and whiter, as the removed dust had a warm grey tone. Therefore, its removal resulted in a whiter and cooler color, characteristic of the paper in this artwork. This was also reported in the literature [30].

Spots 4, 5, and 6 represented measurements taken on the black ink. Spots 4 and 6 were locations of completely opaque ink. In contrast, spot 5 was a reading in an area of lower ink intensity, with a more greyish tone.

**Figure 8:**
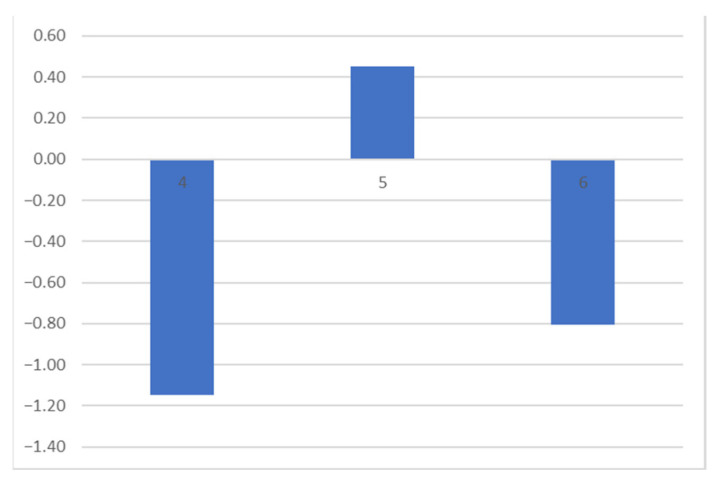
Spots 4, 5, and 6: difference in L* in measurements on black ink, before and after cleaning.

On the black ink, changes in hue were minimal and negligible (see Figure 6). The major change was observed in luminosity, the L* variable. Areas with a high concentration of black ink appeared darker after cleaning because the grey of the dust was removed, revealing the deep black of the ink. The difference in L* plotted in Figure 8 displays this change for spots 4 and 6, which showed a darkening after cleaning due to the appearance of the black inner ink, which, before cleaning, was under a layer with a brownish-greyish tone. In areas with a lower amount of black ink and, therefore, a more greyish tone in the engraving (area 5), the behavior was more similar to that obtained on paper, as the dust was darker than the ink. As observed during the microscopy analysis, areas with grey ink and a lower concentration of black ink became lighter. Thus, spot 5 located in Figure 3 shows a positive difference in the L* measurements (refer to Figure 8).

In terms of the technique’s performance and its practical application in treatment development, there are several factors that should be considered to ensure optimal results. During a cleaning intervention, it is necessary to adjust the cleaning intensity dynamically based on the conservator’s decisions during the treatment. This adjustment should consider various factors, such as the composition, structure, and condition of the area being cleaned, as well as the nature and characteristics of the surface dirt, its interaction with the artefact, and the risk it poses in the short and long term.

A priori, the cleaning intensity can be adjusted by modifying several parameters, including pressure, distance, time, and working angle. However, the effects of cleaning vary depending on these variables.

Changing the pressure is difficult to carry out dynamically, especially because the conservator would have to look away from the cleaning zone to the instrument’s command, which would enable changes in the pressure but involves the loss of visual control during the operation. On the other hand, adjusting the instrument’s regulator to change the pressure can result in significant and sometimes abrupt changes. This can lead to overly aggressive cleaning in certain areas.

As previously stated, altering the working angle affects the cleaning mechanism of the particles upon collision with the surface. Controlling the intensity of the cleaning and the area affected is dependent on the angle of modification. A change in the angle results in an unpredictable variation in cleaning intensity. When the angle of impact is closer to the vertical, the cleaning becomes more aggressive. On the one hand, a sharper working angle results in a more elliptical collision zone for the particles, making it more challenging to control the area being cleaned. On the other hand, a pressure of 20 kPa allows for comfortable cleaning. The energy with which the particles reach the surface is usually sufficient to remove common dirt from paper works, while keeping the hand comfortably resting on the work surface. Adjustments are made by slightly moving the hand backwards from the work. This movement is almost intuitive and does not distract from the view of the work or the concentration needed during the operation.

To adjust the cleaning intensity, time is another variable that needs to be controlled. Checking the cleaning targets helps with deciding when to stop cleaning each area.

## 4. Conclusions

The results of this study indicate that powdered cellulose microblasting is a dry-cleaning technique that, when applied with the appropriate parameters, may be highly effective in cleaning printed works on paper.

In this study, the selected artwork was cleaned efficiently according to the control parameters used to make this assessment. On the one hand, it did not cause morphological changes of the surface, as observed from the optical and scanning electron microscope results. Additionally, both microscopic techniques confirmed that powdered cellulose could be easily vacuumed from paper, even when porous and with an open structure, as well as from lithographic inks, as no residues were detected on the artefact after cleaning.

Through optical and electronic microscopy, it was determined that the level of cleanliness achieved was good, as there has been remarkable remove of soiling, both of free particles and soil imbedded within the fibre’s spaces. This has had a visual effect, both detectable with optical microscopy and spectrophotometry. Dark and cold colours became darker and cooler after cleaning recovering their own nature. In spectrophotometry this is expressed in a decrease of the L*, a* and b*. Other colours or tones appear more vivid when observed with optical microscopy. For example, spectrophotometry revealed an increase in the L* variable in visually grey tones, even when these were low concentration of black ink. In non-inked areas the paper becomes whiter and less reddish-yellow, as the removed soil has a warm grey tone. This is confirmed by spectrophotometry analyses that reveal an increase in brightness of the non-inked paper, confirming the effectiveness of the cleaning method.

The cleanliness control was easy and effective, significantly reducing the treatment time and using materials with a composition compatible with the substrate. After conducting this study, it was concluded that varying the distance and time during the intervention while keeping the pressure constant at 20 kPa and the working angle at 70° is advisable. Additionally, it was found that the working position for cleaning using this technique and these parameters is comfortable, allowing for a full day of work. Furthermore, the technique reduces treatment time compared to commonly used dry cleaning methods for works on paper.

The high long-term stability of cellulose suggests that there will be no harmful effects over time. This has been the case in previous natural ageing studies. Additionally, the compatibility of cellulose with the treated work and the absence of morphological changes and cleaning material residues on the surface suggest that this method will not result in physical, chemical, or mechanical changes. However, further research could explore this topic in more detail.

On the basis of the above, it was confirmed that the microblasting of cellulose powder can be considered a feasible and efficient technique for the dry cleaning of printed works on paper. However, the conservator is the only one who can decide and assess the suitability of any tool, material, or procedure for any specific situation. Therefore, it is plausible that this method, like any other, may not be appropriate for all graphic document works and in all contexts.

This research suggests that microblasting of powdered cellulose is an advantageous technique in comparison to the methods commonly used in dry cleaning paper-based works. The studied technique mitigates the proven negative effects and foreseeable ones that may be caused by the poor stability of the used cleaning materials and their low compatibility with the original artefacts.

## Data Availability

Data are contained within the article.
